# Inverse design of an ultra-compact broadband optical diode based on asymmetric spatial mode conversion

**DOI:** 10.1038/srep32577

**Published:** 2016-09-02

**Authors:** Francois Callewaert, Serkan Butun, Zhongyang Li, Koray Aydin

**Affiliations:** 1 Department of Electrical Engineering and Computer Science Northwestern University, 2145 Sheridan Road, Evanston, IL 60208, USA.

## Abstract

The objective-first inverse-design algorithm is used to design an ultra-compact optical diode. Based on silicon and air only, this optical diode relies on asymmetric spatial mode conversion between the left and right ports. The first even mode incident from the left port is transmitted to the right port after being converted into an odd mode. On the other hand, same mode incident from the right port is reflected back by the optical diode dielectric structure. The convergence and performance of the algorithm are studied, along with a transform method that converts continuous permittivity medium into a binary material design. The optimal device is studied with full-wave electromagnetic simulations to compare its behavior under right and left incidences, in 2D and 3D settings as well. A parametric study is designed to understand the impact of the design space size and initial conditions on the optimized devices performance. A broadband optical diode behavior is observed after optimization, with a large rejection ratio between the two transmission directions. This illustrates the potential of the objective-first inverse-design method to design ultra-compact broadband photonic devices.

There has been a lot of interest recently for photonic devices that can achieve asymmetric light transmission or optical diode behavior. Typically, an optical diode is a two-port device where light coming from the first port is transmitted to the other port while light coming from the second port is not transmitted to the first port but either reflected, deflected or absorbed instead. In particular, there is high interest for on-chip asymmetric light transmission devices for integrated photonic applications. An optical isolator is the ideal solution as it can transmit and block any spatial mode in the two directions. However, optical isolation is very challenging to be implemented in integrated devices because of the need to break the Lorentz symmetry condition, which means the scattering matrix of such a device must be asymmetric^1^. This can only be achieved with large devices based on magneto-optic materials^2^,^3^,^4^,^5^ or indirect interband transitions^6^ usually not compatible with CMOS fabrication processes.

On the other hand, optical diode behavior can be achieved in a much simpler way with a reciprocal device based on asymmetric mode conversion^7^. Such a device relies on spatial symmetry breaking, which means that it can be done with any type of material. Reciprocal optical diodes can only achieve asymmetric transmission with specific modes^1^. Nonetheless, they typically need much smaller footprint than optical isolators, and some reciprocal diode designs are compatible with CMOS fabrication. Various types of reciprocal optical diodes have been demonstrated recently based on chiral metamaterials^8^,^9^,^10^,^11^,^12^, hyperbolic metamaterials^13^, digital metamaterials^14^, metasurfaces^15^, ring resonators^16^, metal-silicon waveguides^17^ and photonic crystals^18^,^19^,^20^,^21^,^22^,^23^. Particularly, a very compact optical diode was proposed recently based on a photonic crystal structure made of silicon and air^7^. In the reported device, the first even spatial mode from the left waveguide is converted into the first odd spatial mode by the optical diode structure and transmitted to the right waveguide, while the first even mode from the right is reflected back, which does not violate the reciprocity principle. However, the reported device only works with air waveguide and needs to be integrated into a larger photonic crystal medium, which increases the total footprint of the device (~2 × 4 square wavelength). Furthermore, the reported optical diode could only operate around a very small bandwidth, which is ~1% of the resonant wavelength.

Here we report an ultra-compact and broadband optical diode designed with a new computational method to exceed the limitations from photonic crystal structures’ design. We use the concept of “objective-first inverse-design”^24^,^25^,^26^,^27^ developed by Vuckovic *et al*. where the local permittivity of the device area is engineered with an optimization algorithm in order to achieve desired optical diode behavior. The algorithm is adapted in order to design a broadband optical diode with ultra-compact footprint (~1 square wavelength), based on silicon and air medium and integrated between two silicon waveguides.

In this paper, the inverse-design optimization algorithm is firstly introduced with the choice of the objective in order to realize the optical diode behavior with a binary device. The convergence is shown, the performance of the proposed algorithm is discussed and then the optical behavior of the diode is analyzed. Finally, a parametric study is designed to explain the impact of the design space parameters such as size and initial permittivity on the final device’s performance. The performance of 3D devices based on our 2D simulations are also discussed as a function of the thickness.

## Structural design methods

### Optimization algorithm

Objective-oriented photonic device design is not a straightforward task. Typically, one wants to convert a given electromagnetic (EM) wave input into a desired EM output following an “objective” such as spectral modulation or mode conversion. The key is to find the specific dielectric structural distribution of *ε* and the EM fields (*E, H*) in the region of interest that allows the conversion. The main challenge here is that the optimization of *ε*, *E* and *H* at the same time is a highly non-convex problem intractable with traditional numeric methods.

Typically, this problem is divided in two steps with a trial-and-error process. One first specifies a test dielectric structure *ε* and simulates how light interacts with the structure with full-wave electromagnetic simulations. If the device does not behave as expected, one tries a new structure and restarts the process. The search of the optimal dielectric structure relies either on the researcher’s intuition or on algorithms that perform the structure design automatically^28^. Most numerical methods are based either on brute force search^14^, stochastic search or they need a drastic reduction of the design space, for example by using a photonic crystal structure^7^. As a result, they are either extremely computationally intensive or very restrictive on the degrees of freedom of the design. On the other hand, the inverse-design method implements the objective directly in the optimization algorithm that searches for the dielectric structure. As a result, the algorithm converges much faster towards the objective, and it is much less computationally intensive and can manage a large number of degrees of freedom.

In short, the algorithm starts defining the device as a “black box”, a physical space where it will determine the dielectric structure *ε* and the magnetic field *H*_in_. An objective is defined that the device needs to achieve, for example a specific magnetic field *H*_bnd_ at the boundaries. The algorithm then starts optimizing the structure and field in order to match the objective exactly (objective-first algorithm) and follow the electromagnetic wave equation as closely as possible. The algorithm alternates two steps. One step takes *ε* as input and determines *H* which minimizes the residual in Maxwell equations, which we call the “physics residual” following Vuckovic’s notation^26^. Mathematically, we have:


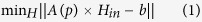


where 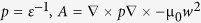 and 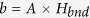 is the “source”.

The other step is very similar but reverses the roles of *p* and *H*:


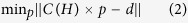


where 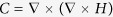 and 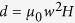.

Both steps rely on convex optimization, and the CVX Matlab package is used to specify and solve them^29^,^30^. By alternatively performing these two sub-problems many times, the algorithm converges towards a dielectric structure which is expected to behave closely to specified by the objective. Much greater details on the method can be found in Lu *et al*.^26^. Here we present the objective used for the optical diode.

**Figure 1:**
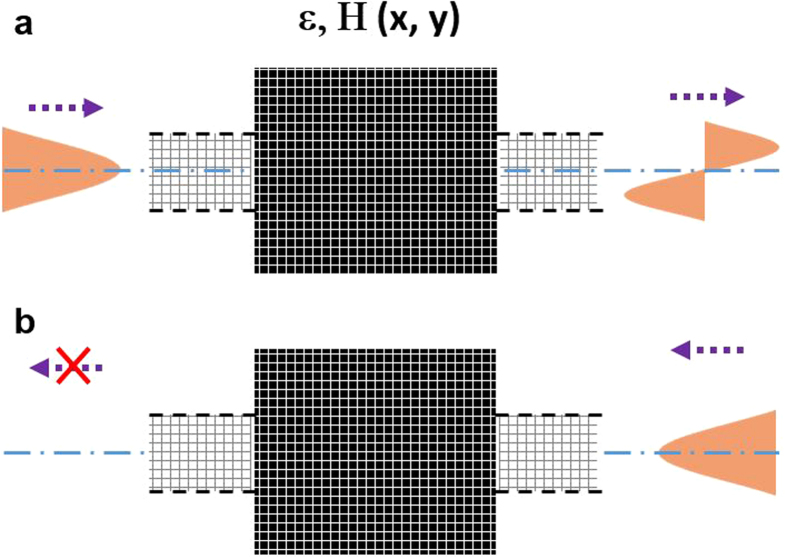
Schematic representation of the optical behavior for an optical diode. The first even mode is fully transmitted when it comes from the left port (**a**) and converted to the odd mode. Same input is reflected back when coming from the right port (**b**).

The black box behaves as a converting structure between two wave-guiding ports. The objective is given by specifying the magnetic field at the boundaries of the box. The first objective is that, when light transmits from left to right (L → R), the EM field at the left boundary corresponds to the first even mode of the left port and the field at right is the first odd mode of the right port (as shown in Fig. 1a). Additionally, the second objective is that, when the first even mode is incident from the right port (R → L), the field at the right boundary should be a superposition of the first even mode of incident light and reflected light, and the field on the left port should be null to achieve zero transmission (as shown in Fig. 1b). It is worth noting that the desired field profile at the top and bottom boundaries should be zero in order to prevent any optical power loss outside of the device.

Compared to a simple inverse design algorithm with one condition, we only modify the size of the field variable *H* and the “source” *b*, which both become a *N* × 2 matrix instead of a *N*-vector, where *N* is the number of pixels in the device and 2 is the number of conditions. In general, this method can be used to assign any number *k* of objectives that one wants the device to achieve using a *N* × *k* matrix, with a computational cost proportional to *k*.

### Conversion to binary structure

One issue of the inverse design algorithm is that the permittivity of the resulting structure is distributed to a continuous function of values, for instance 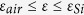. However, a practical photonic device is fabricated with a discrete set of materials, often two materials only such as silicon and air. Unfortunately, convex optimization can only be performed with a continuous set. Another algorithm is needed in order to convert the structure obtained after optimization into a binary structure. The easiest approach is to define an intermediate permittivity value 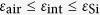 such that the algorithm creates a binary structure *ε*_bin_ from *ε* such that, for each pixel i:


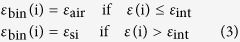


The intermediate value 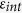 can be chosen in order to minimize the physics residual of the resulting binary structure. However, if the original structure is not already close to binary, *ε*_bin_ is expected to differ significantly from it. As a result, the binary structure will most likely behave differently and have a poor performance.

In order to favor the convergence of the optimization algorithm towards a binary structure, we add a “binarization” cost to Equation (2) proportional to the difference between the structural variable p and the binary value p_bin_ obtained at the previous iteration, which is a cost linear in p, thus convex. With this additional cost, Equation (2) is replaced by:


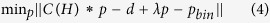


Where *λ* is a constant to adjust the relative “strength” of the binarization: *λ*  = 0 is the “optimization” case while *λ* = ∞ is the binary case. The best results are achieved starting with *λ*  = 0 and progressively increasing it. By making the optimized structure close to binary, this algorithm will ensure that the performance is not impacted too much when the device is converted to a purely binary structure.

### Simulation procedure and figures of merit

In this work, simulations are performed in a two dimensional space, assuming Transverse Electric field (TE), so the magnetic field *H* is perpendicular to the simulation surface. The permittivity values for the local medium is set to vary between *ε*_*min*_ = 1 (for the air) and *ε*_*max*_ = 12 (for silicon). For every iteration of the algorithm, we solve Equation (1), then Equation (2). We also perform two Finite-Difference Frequency Domain (FDFD) simulations to evaluate the performance of the device (the transmitted power) when the first even mode is incident from each side of the ports.

The designs are evaluated according to four figures of merit. The physics residual R corresponds to the minimized value in Equation (1) and (2). The most important figure of merit is the transmission, which is the ratio of the output power to the input power and which is computed for both directions *T*_*LR*_ (left to right) and *T*_*RL*_ (right to left). Finally, the binary coefficient *B* characterizes how close the computed continuous distribution of *ε* is to a binary structure, which is mathematically defined as:


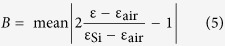


*B* = 1 when the structure is purely binary and *B* = 0 when the structure’s permittivity is uniformly distributed halfway between the air and silicon’s permittivity values. This figure of merit is used to evaluate how well the binarization algorithm behaves when compared to a simple optimization.

For every iteration, the figures of merit of both the structure with continuous *ε* (the “continuous structure”) and the binary structure are computed, the latter being the one that matters for a practical device and thus the one that we want to optimize.

## Inverse-design of an optical diode

First, we study the optimization process while designing an optical diode using the binarization algorithm that solves equations 1 and 4. The design space size is chosen to be 3/4*λ* × 3/2*λ* where *λ* is the wavelength, and the resolution is 40 pixels/*λ* (30 × 60 pixels). The waveguide width is 0.4*λ* (16 pixels) and the initial permittivity in the device is uniformly equal to 8*ε*_0_.

### Algorithm convergence

**Figure 2:**
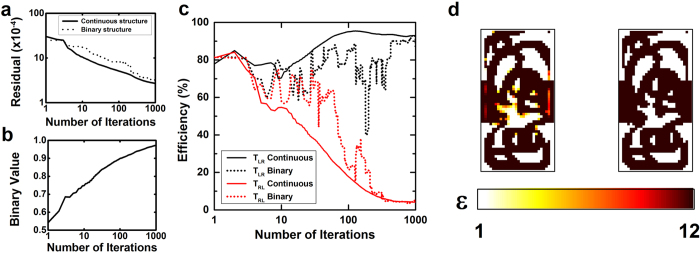
Physics residual (**a**), binary value (**b**) and transmission efficiencies for both direction incidences (**c**) of the designs computed by the objective-first inverse design algorithm as a function of the number of iterations. Figures of merit from the continuous and binary structures are respectively shown in solid and dotted lines. (**d**) Color maps of the continuous (left) and binary (right) structures calculated by the inverse-design algorithms after 1000 iterations. Each structure is composed of 30 × 60 pixels, where the color shows the computed permittivity, between 1 (air) and 12 (Silicon).

The algorithm is run for 1000 iterations with the parameters given above. The evolution of the figures of merit is reported in Fig. 2 as a function of the iteration number. As expected, the physics residual field decreases (solid black curve in Fig. 2a) along the optimization process. This results in the transmission efficiencies getting closer and closer to the ideal optical diode values, with a final transmission ratio *R*_*cont*_* = T*_*LR*_*/T*_*RL*_ = 92.6%/2.8% = 33 (30 dB). Thanks to the binarization cost, the continuous structure converges quickly towards a structure close to binary, as shown by the evolution of the binary value (Fig. 2b) that converges towards 1. As a result, both the residual and the transmission efficiencies of the binary structure (dotted curves in Fig. 2a,c) follow closely the values of the continuous structure, with a very similar final transmission ratio of *R*_*bin*_ = 93.5%/3.2% = 29 (29 dB).

The continuous and binary designs computed are represented by the color maps of Fig. 2d and their respective figures of merit are shown in Table 1.

### Analysis of the structure and field profile

As discussed in previous part, the optimal binary structure achieves a performance very close to the ideal optical diode. Remarkably, this performance is achieved with a design area of only one square wavelength, which is one of the smallest optical diode reported to date, and in particular 10 times smaller than the performance of the photonic crystal based optical diode reported in ref. 7.

**Figure 3:**
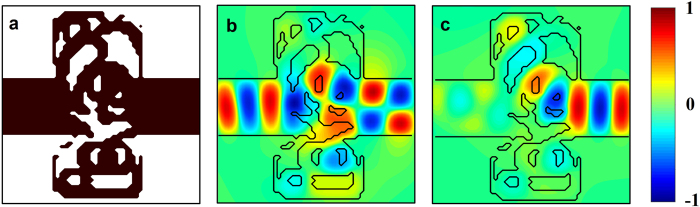
(**a**) Color map of the optimal binary dielectric structure (4), black being Silicon and white is air. (**b,c**) are color maps of the real part of the magnetic field in the optical diode as calculated from FDTD simulations under excitation either from the left waveguide (**b**) or from the right waveguide (**c**).

The structure of the optical diode and its operation are represented in Fig. 3. Figure 3a is a color map of the permittivity distribution, where black corresponds to silicon and white is air. Figure 3b,c are color maps of the real part of the magnetic field *H* when the first even mode is incident from the left and right ports, respectively. In the case of left-to-right transmission, the even mode from the left is converted into an odd mode to the right as expected. This mode conversion is achieved by adding a 2π phase to the part of the field going through the lower part of the structure, as opposed to a 3π phase change for the part of the field going through the upper part. On the other hand, when the mode is sent from right to left, most of the power is deflected towards the top, then reflected back by the successive silicon-air layers that act as a Bragg mirror.

Color map animations of the magnetic field evolution in the optical diode under fundamental spatial mode excitation from the left and from the right waveguide are provided as a supplementary information.

### Wavelength-dependence characteristics

In order to check our results and study the wavelength dependence of our optical diode’s performance, a commercially available numerical solver was used to perform finite-difference time-domain (FDTD) simulations of the proposed structure. The size of the structure is chosen so the operation wavelength is 1550 nm, but any resonant wavelength could be chosen by simply scaling up or down the structure.

**Figure 4:**
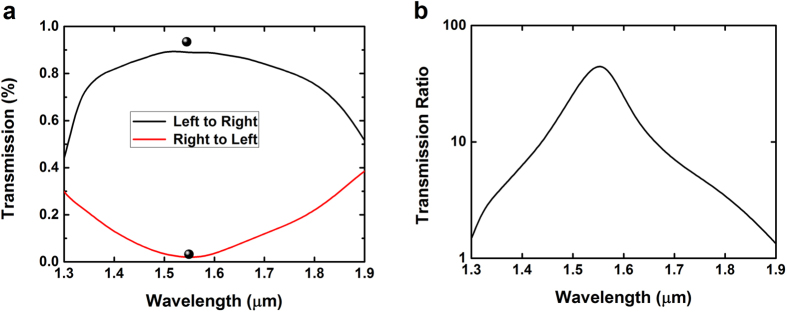
(**a**) FDTD simulations of the optical power transmission in the optical diode as a function of the wavelength in both directions. The spheres represent the values calculated by our FDFD algorithm. (**b**) Ratio between the two transmissions.

FDTD simulations are firstly performed in 2D settings, with a mesh size equal to half a pixel. The structure of Fig. 4 is built out of silicon (*ε* = 12), with air as the surrounding medium (*ε* = 1), and we use stretched coordinates PML boundary conditions. A pair of simulation are performed and compared: one using a source which is the first even mode incident from the left port with TE polarization and the other which is the same source but incident from the right port. The transmission as a function of the wavelength is plotted for both directions in Fig. 4a. As can be seen, FDTD simulations (lines) agree very well with simulations from our FDFD code (spheres) at the resonant wavelength. Furthermore, we can see that the optical diode behavior is covering a broad wavelength spectrum. The left-to-right transmission stays higher than 80% and the right-to-left transmission stays lower than 10% for wavelengths between 1.4 and 1.7 μm. This corresponds to a large relative spectral width Δ*λ*/*λ*  = 20% as opposed to the very narrow spectral width of a photonic crystal based optical diode of 1–2% only^7^. A ratio of up to 45 (33 dB) is observed between the two transmissions at resonant wavelength.

## Parametric study

The designs obtained with our algorithm are highly dependent on the simulation parameters such as the size of the black box or the initial conditions. Here we show a parametric study to understand the process of finding an optical diode with the best tradeoff between performance (transmission ratio), compactness, and manufacturability (thickness). Although the design part is exclusively done in 2D, we will also discuss about how well the performance can be reproduced with 3D designs.

### Simulation parameters

The algorithm is run on a rectangular grid of pixels, defined by the pixel size chosen to be equal to *λ*/40 for this study, the length *L* and width *W* all expressed as a function of the wavelength *λ* in the vacuum. The waveguiding ports width is chosen to be ~0.4*λ*. Finally, the initial value of the permittivity 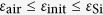 is set to be uniform in the black box.

### Design space size

**Figure 5:**
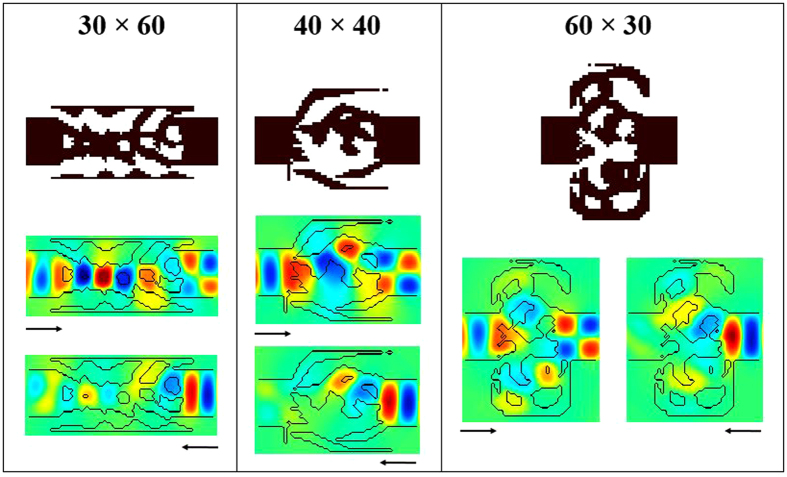
Color map of the dielectric structures (black and white) and the real part of the magnetic field (color) in the three optical diodes with aspect ratio 1:2 (left), 1:1 (middle) and 2:1 (right).

We first study how the shape and size of the design space impact the device performance. For all the designs in this part, the initial permittivity is *ε*_*init*_ = 4. First, we study the impact of the design space aspect ratio on the performance while keeping the device area constant, approximately equal to *λ*^2^. The three aspect ratio tested are *W:L* = 1:2, 1:1 and 2:1, for a number of pixels of 30 × 60, 40 × 40 and 60 × 30 respectively. The algorithm is run for each design space, and the binary devices’ performances after optimization are summarized in Table 2. The structures and the magnetic field under excitation from the left waveguide are also shown in Fig. 5.

As can be seen, the performance of the long structure (*W:L* = 1:2) is quite low, with a transmission ratio of only 6. From the field profile, we see that only the right part of the structure acts as an optical diode while the left part is mostly wasted space extending the left waveguide. On the other hand, the square and wide structures both show good transmission ratio. This indicates that the choice of the design space is critical for the algorithm to converge towards a good design. Here, the wide structure aspect ratio is preferred for the best optical diode performance.

**Figure 6:**
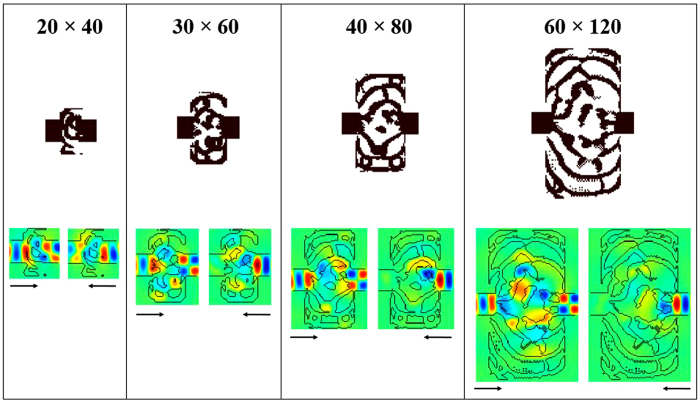
Color map of the dielectric structures (black and white) and the real part of the magnetic field (color) in the four optical diodes with, from left to right, size W × L = 20 × 40, 30 × 60, 40 × 80 and 60 × 120.

Next, we study the influence of the design space size for a fixed aspect ratio chosen to be *W:L* = 2:1 based on the previous optimization. Four device sizes are successively tested, with *W* × *L* = 20 × 40, 30 × 60, 40 × 80 and 60 × 120 respectively from the smallest to the largest design, with respective device areas of *λ*^2^*/2, λ*^2^*, 2λ*^2^ and *4λ*^2^. The binary structures and fields are represented in Fig. 6 and the performance in Table 3. As expected, the performances of the first three designs improve with the size as more and more degree of freedoms are available for the algorithm to converge towards better solutions. However, the performance does not improve anymore when the design space is too large (60 × 120), mainly because the binary conversion is less precise.

For this study, we also report the performance of 3D structures based on the computed 2D designs but with a finite thickness equal to *λ/4* and surrounded by air. As can be seen, the transmissions of the 3D designs are usually much lower than the 2D cases, especially in larger structures. This is mainly because in thin structures the field is not confined efficiently and “leaks” from the top and bottom of the structure, particularly in the air gaps of the silicon structure. The leakage is proportional to the total area of the gaps, which explains why the transmission is so low in large structures. For this reason and although the 2D performance is not optimal, we prefer the smaller 30 × 60 structure that is expected to have better performance in a practical device.

### Initial permittivity

**Figure 7:**
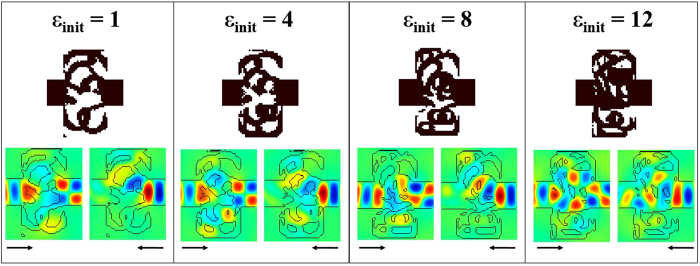
Color map of the dielectric structures (black and white) and the real part of the magnetic field (color) in the four optical diodes computed starting from an initial permittivity *ε*_*init*_ = 1, 4, 8 and 12 from left to right.

Now that the design space size has been optimized, we study the impact of the initial conditions on the design, specifically the initial permittivity that is used at the first iteration of the algorithm. For this study, the device size is *W* × *L* = 3/4 *λ*  × 3/2 *λ* (30 × 60 pixels). The binarize algorithm is run for initial integer permittivities between 1 and 12. Here we report the optimized structures for *ε*_*init*_ = 1, 4, 8 and 12 respectively. The binary structures and fields are represented in Fig. 7 and the performance of both the 2D and 3D structures with thickness equal to *λ/4* are shown in Table 4. First of all, we notice that the algorithm converges towards very different structures depending on the initial conditions. This shows that the inverse-design algorithm is not designed to converge towards a global optimum, but rather towards local optima. Depending on the initial permittivity, the final structure will be composed of air for the most part if *ε*_*init*_ is small and of silicon for the most part when *ε*_*init*_ is large. From the perspective of the field, the optical diode behavior can be achieved in many ways. One way to understand intuitively the difference between these structures is by visually finding the phase change along the shortest optical path between the left and right waveguides. In the case of left to right transmission, the smallest phase change is π when *ε*_*init*_ = 1 or 4, 2π when *ε*_*init*_ = 8 and 3π when *ε*_*init*_ = 12. As for the performance, we can see that the first three designs have very similar transmissions in 2D although the structures differ drastically. Nonetheless, the performances of the 3D structures are very different and seem to favor structures with more silicon, as the third structure has a much better transmission from left to right. The reason is that the field is better confined vertically in structures with a high silicon content, which prevents scattering of optical power from the bottom and top of the structure. However, too much silicon content does not allow an efficient optical diode behavior as well. Empirical observation show that the best 3D performance is achieved with *ε*_*init*_ ≈ 7–8 *ε*_*0.*_

### Device thickness in a three dimensional structure

**Figure 8:**
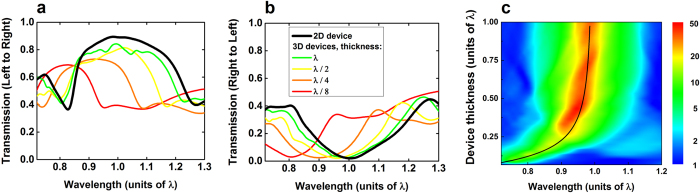
FDTD simulations of the optical power transmissions (**a**,**b**) and ratio (**c**) in a 3D optical diode etched in a SOI wafer as a function of the wavelength and for various device thicknesses.

So far we studied the performance of 2D structures, which is equivalent to considering infinitely thick structures in 3D, and also the performance of *λ*_0_*/4*-thick devices surrounded by air, where *λ*_0_ is the target resonant wavelength set in equations (1) and (2). These results are extended here to the case of a more realistic structure with finite thickness etched through a silicon-on-insulator (SOI) wafer. The structure is assumed to be etched through the entire upper silicon layer, until the insulator material which is assumed to be silicon dioxide for the calculations. The performance of 3D devices based on the design from Fig. 3 are studied as a function of the thickness. The transmission in both directions are shown in Fig. 8a,b for device thicknesses of *λ*_0_, *λ*_0_/2, *λ*_0_/4 and *λ*_0_/8 as well as for an infinitely thick device (2D). Figure 8c is a color map representing the transmission ratio as a function of the wavelength and the device thickness both expressed as a function of *λ*_0_. As can be seen, the thinner the 3D device, the higher the difference with the 2D case. In particular, the resonant wavelength *λ* is blue-shifted when the device thickness t decreases, following the empirical law:


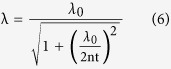


Where *n* is an empirical coefficient equal to 6 for the case of this device. This empirical law is represented by the black line in Fig. 8c. Although the transmission ratio and bandwidth decrease with the thickness, very good optical diode performance can be achieved for devices as thin as *λ*_0_/6, with a transmission ratio higher than 10 over a large bandwidth Δ*λ*/*λ* ≈ 13%. Such an optical diode optimized for telecom wavelengths (around 1550 nm) would yield a device with an area of approximately 3 μm^2^ and a thickness of 250 nm, which corresponds to most common SOI wafers commercially available and should be easy to fabricate with current silicon processes.

## Conclusion

An ultra-compact optical diode based on silicon and air only has been designed with the objective-first inverse-design algorithm. The device operates by converting the first even mode from the left port into the first odd mode of the right port, while the first even mode from the right port is reflected back by the structure. Only a few minutes of computing time are needed in order to converge towards a high performance design, which means one can test the influence of various parameters very fast. A parametric study was designed in order to understand and optimize the impact of the design space size and initial permittivity on the final designs. We found that although large design spaces allow convergence towards very high performance structures in 2D, smaller designs behave better in practical 3D devices due to the lower scattering, and devices with higher silicon content are preferred for the same reason. Optimized design shows a broadband optical diode behavior around the resonant wavelength, with a peak transmission ratio of 30 between excitations from the left and right sides. Compared to previous silicon-air optical diodes devices, mostly based on photonic crystals, this design has a smaller footprint of 1 square wavelength and a broader wavelength spectrum. More generally, the inverse-design method is a very powerful tool to automate the design of extremely efficient photonic devices with very small footprint.

## Additional Information

**How to cite this article**: Callewaert, F. *et al*. Inverse design of an ultra-compact broadband optical diode based on asymmetric spatial mode conversion. *Sci. Rep.* **6**, 32577; doi: 10.1038/srep32577 (2016).

## Supplementary Material

Supplementary Information

Supplementary Image 1

Supplementary Image 2

## Figures and Tables

**Table 1:** Figures of merit of the 2 structures in Fig. 2d.

Algorithm	Residual ( × 10^−4^)	Binary	Transmission L → R	Transmission R → L
**Continuous structure**	2.8	0.97	92.6%	2.8%
**Binary structure**	3.3	1	93.5%	3.2%
**Ideal value**	0	1	100%	0%

**Table 2:** Performance (transmissions and ratio) of the binary structures in Fig. 5.

Structure Size (W × L)	T_L → R_	T_R → L_	Ratio
30 × 60	64.1	10.4	6
40 × 40	85.8	4.1	21
60 × 30	90.2	3.4	27

**Table 3:** Performance (transmissions and ratio) of the binary structures in Fig. 6.

Structure Size (W × L)	2D T_L → R_	2D T_R → L_	2D Ratio	3D T_L → R_	3D T_R → L_	3D Ratio
**20 × 40**	81.8	37.3	2.2	75	30	2.5
**30 × 60**	90.2	3.4	27	57	1.8	32
**40 × 80**	95.6	0.6	159	37	0.9	41
**60 × 120**	93.6	1.0	94	17	0.18	94

**Table 4:** Performance (transmissions and ratio) of the binary structures in Fig. 7.

Initial permittivity	2D T_L → R_	2D T_R → L_	2D Ratio	3D T_L → R_	3D T_R → L_	3D Ratio
**1**	90.3	4.1	22	52	2.2	24
**4**	90.2	3.4	26	57	1.8	32
**8**	93.5	3.2	29	78	2.6	30
**12**	83.5	27.1	3.1	72	21	3.4
