# Supplementary material for: Inverse design of an ultra-compact broadband optical diode based on asymmetric spatial mode conversion
Source: Sci Rep. 2016 Sep 2;6:32577. doi: 10.1038/srep32577 (PMC5009310; doi:10.1038/srep32577)
Supplement: Supplementary Information [file srep32577-s1.doc]

**Supplementary Information for:**

**Inverse design of an ultra-compact broadband optical diode based on asymmetric spatial mode conversion**

**F. Callewaert, S. Butun, Z. Li and K. Aydin**

*Department of Electrical Engineering and Computer Science*

*Northwestern University, 2145 Sheridan Road, Evanston, IL 60208*

Two animations:

**Supplementary file 1** **(Left to Right.gif)**: Color map animation of the magnetic field evolution in the optical diode under fundamental spatial mode excitation from the left waveguide.

**Supplementary file 2** **(Right to Left.gif)**: Color map animation of the magnetic field evolution in the optical diode under fundamental spatial mode excitation from the right waveguide.
