# Supplementary figures and images for: Inverse design of an ultra-compact broadband optical diode based on asymmetric spatial mode conversion
Source: Sci Rep. 2016 Sep 2;6:32577. doi: 10.1038/srep32577 (PMC5009310; doi:10.1038/srep32577)

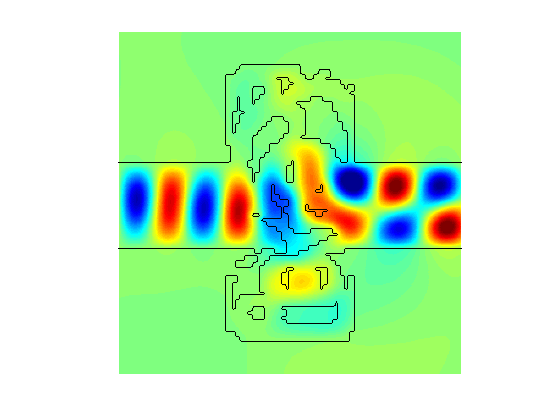

Supplement: Supplementary Image 1 [file srep32577-s2.gif]

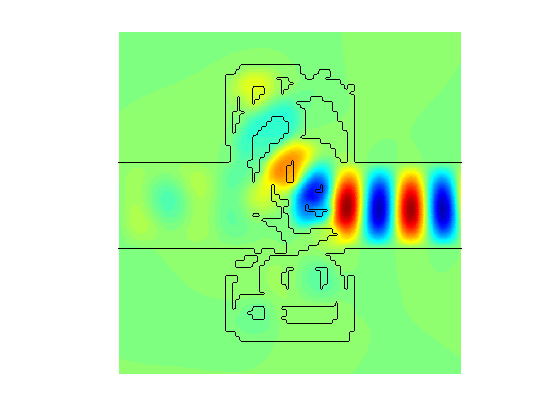

Supplement: Supplementary Image 2 [file srep32577-s3.gif]
